# Postoperative cutaneous metastasis of breast cancer with phenotypic conversion to triple-negative subtype: A case report

**DOI:** 10.1097/MD.0000000000050166

**Published:** 2026-08-14

**Authors:** Shucheng Zhang, Xiaoyue Sun, Zhuqing Li, Shijuan Wei, Xiaoqing Si

**Affiliations:** a Shandong First Medical University, Jinan 250117, China; c Shandong University of Traditional Chinese Medicine, Jinan 250355, China; d Shandong Second Medical University, Weifang 261053, China; b Department of Dermatology, The First Affiliated Hospital of Shandong First Medical University, Jinan 250013, China

**Keywords:** breast cancer, cutaneous metastasis, phenotypic conversion, triple-negative

## Abstract

**Rationale:**

Cutaneous breast cancer metastasis carries poor prognosis. Phenotypic conversion to a triple-negative subtype presents therapeutic challenges. Re-biopsy is crucial.

**Patient concerns:**

A 53-year-old female underwent right modified radical mastectomy for HER2-positive invasive breast carcinoma in September 2023, and developed pruritic, erythematous nodules on the right chest wall, axilla, and back 9 months later.

**Diagnoses:**

Histopathology revealed poorly differentiated dermal carcinoma. The primary tumor was ypT2N3aMx with 23/23 positive nodes, grade 3. IHC showed ER(-), PR(-), HER2 (3+), and Ki-67 (~40%). In October 2023, a supraclavicular lymph node biopsy revealed HER2 IHC 0, representing the first evidence of phenotypic conversion. In July 2024, biopsy of the cutaneous metastatic lesion confirmed HER2 IHC 0 (no membrane staining), ER(-), PR(-), and Ki-67 (~60%+), consistent with a persistent triple-negative phenotype.

**Interventions:**

Treatment was switched to gemcitabine plus cisplatin combined with envafolimab (a PD-L1 inhibitor). Prior to initiation, PD-L1 IHC (SP263, Ventana) revealed TC 0%, IC+ <1%, and ICP ~10%. Six cycles were administered between August and November 2024. In January 2025, new lesions with ER 20% positivity emerged, and the regimen was adjusted to vinorelbine plus bevacizumab combined with envafolimab.

**Outcomes:**

The patient completed 6 cycles of gemcitabine plus cisplatin combined with envafolimab. Course was uneventful, with isolated grade 3 neutropenia (0.71 × 10^9/L) in October 2024. In January 2025, new cutaneous lesions emerged, prompting biopsy and regimen change. As of April 2025, the patient remained on active treatment with stable disease status.

**Lessons:**

This case underlines re-biopsy importance. Conversion to a triple-negative phenotype necessitates complete change in systemic therapy. Phenotypic conversion may first manifest in lymph node metastases before becoming clinically evident in cutaneous lesions.

## 1. Introduction

Breast cancer is the most common malignancy among women globally, with recurrence and metastasis being the primary causes of treatment failure.^[1]^ The skin is a recognized but relatively uncommon site for breast cancer metastasis, often indicating advanced disease and a poor prognosis. Bone, liver, lung, brain, and lymph nodes represent more typical distant metastatic sites.^[1]^ Breast cancer is highly heterogeneous, and its molecular classification based on the expression of estrogen receptor (ER), progesterone receptor (PR), and human epidermal growth factor receptor 2 (HER2) serves as the cornerstone for guiding targeted therapy and predicting outcomes.^[2]^

However, the molecular phenotype of breast cancer is not static. Clinical studies have confirmed that significant discrepancies in receptor expression can exist between the primary tumor and metastatic lesions, a phenomenon known as “phenotypic conversion.” A molecular analysis of cutaneous metastases from breast cancer found that the triple-negative phenotype accounted for 36.4% of cases, and nearly half (48.5%) of the patients exhibited molecular alterations in their skin metastases that differed from the primary tumor.^[2]^ This conversion can render treatment strategies based on the primary tumor ineffective, particularly when a HER2-positive tumor transforms into a triple-negative phenotype lacking effective therapeutic targets, posing a significant clinical challenge.^[3]^ Research indicates that tumor cell phenotypic plasticity, such as heterogeneity driven by epithelial-mesenchymal transition (EMT), may be one mechanism contributing to therapeutic adaptation and metastasis.^[4]^ There is even evidence that HER2-positive breast cancer may transiently shift to other subtypes under the selective pressure of targeted therapy.^[5]^ Therefore, re-biopsy of recurrent or metastatic lesions to reassess biomarkers is critical. We report a case of HER2-positive breast cancer in which phenotypic conversion to a triple-negative phenotype was first identified in a supraclavicular lymph node metastasis and subsequently confirmed in postoperative cutaneous metastases, highlighting the clinical challenges posed by such phenotypic discordance.

## 2. Case report

A 53-year-old female presented to our department in July 2024 with a one-month history of scattered red nodular elevations on the right chest wall, axilla, and back. Physical examination revealed absence of the right breast with a surgical scar on the right chest wall. Dermatological examination showed large, dusky red patches on the right chest wall, back, and axilla. The surfaces of these patches were coalescing and studded with verrucous, firm nodules (Fig. 1).

**Figure 1.**
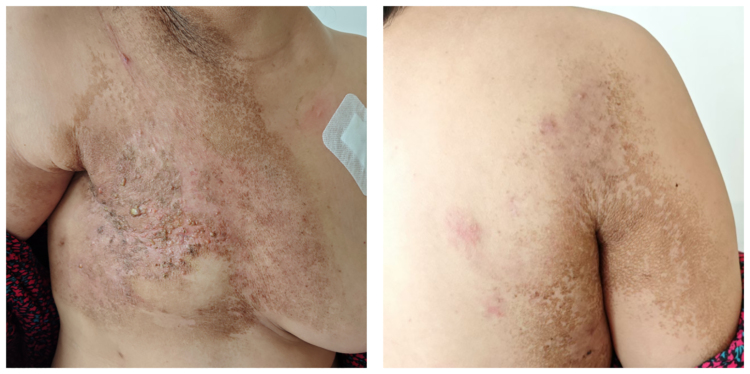
Clinical presentation of cutaneous metastases. Large, dusky red patches studded with verrucous, firm nodules are observed on the right chest wall, axilla, and back.

In January 2023, the patient had discovered a right breast mass (approximately 1.5 × 1.0 cm). In April 2023, she was diagnosed with invasive breast carcinoma. Immunohistochemistry (IHC) of the core needle biopsy showed ER(−), PR(−), HER2 (3+), and Ki-67 (~40%). She received neoadjuvant chemotherapy comprising cyclophosphamide and epirubicin for 2 cycles, followed by paclitaxel liposome combined with cyclophosphamide and epirubicin for 2 cycles, and subsequently paclitaxel liposome combined with trastuzumab and pertuzumab for 2 cycles. In September 2023, she underwent a right modified radical mastectomy with axillary lymph node dissection under general anesthesia. The postoperative pathology confirmed invasive carcinoma of no special type (ypT2) of the right breast (Figs. 2A, 2B). The tumor extensively involved the axillary and interpectoral lymph nodes (23/23 positive), staging the disease as ypT2N3aMx. IHC of the primary tumor showed: ER(−), PR(−), HER2 (3+), Ki-67 (~40%) (Fig. 3A–C). Postoperative adjuvant therapy comprised paclitaxel liposome plus carboplatin combined with trastuzumab and pertuzumab for 2 cycles.

**Figure 2.**
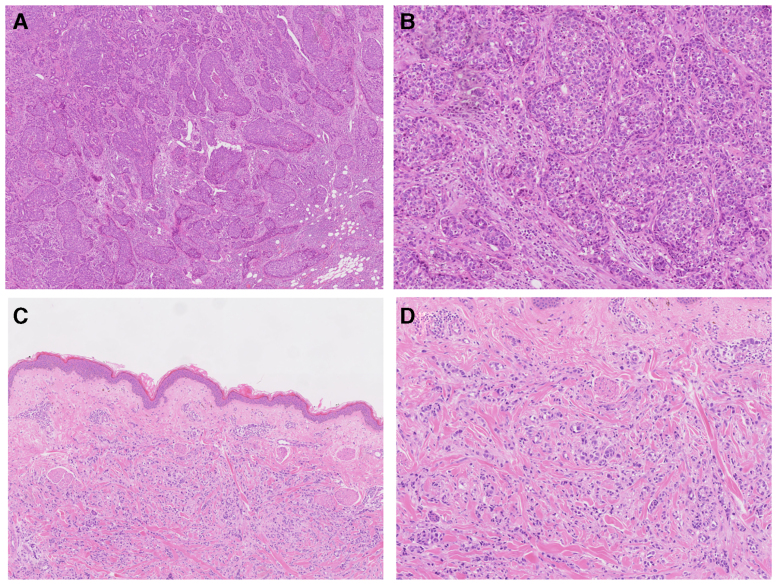
Histopathological comparison (H&E staining). (A, B) Primary invasive carcinoma of no special type in the breast tissue (A: ×50, B: ×200). (C, D) Metastatic, poorly differentiated carcinoma within the dermis, confirming cutaneous spread from breast primary (C: ×50, D: ×200).

**Figure 3.**
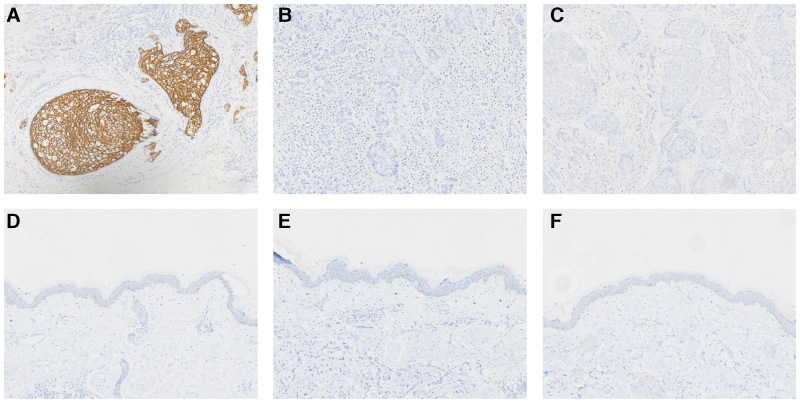
Immunohistochemical confirmation of phenotypic conversion. Primary tumor (A–C): HER2 3 + (A), ER negative (B), PR negative (C). Cutaneous metastasis (D–F): HER2 negative (D), ER negative (E), PR negative (F). All images × 200. The loss of HER2 expression confirms conversion to a triple-negative phenotype.

In October 2023, the patient was found to have right supraclavicular lymph node metastasis. A biopsy of the supraclavicular lymph node revealed metastatic adenocarcinoma with IHC showing ER (weakly positive, ~1%), PR(−), HER2 IHC 0, Ki-67 (~70%), GCDFP-15 (focally positive), mammaglobin (focally positive), and GATA3 (+). This represented the first evidence of phenotypic conversion from the primary HER2-positive tumor to a HER2-negative phenotype. The treatment regimen was subsequently adjusted to trastuzumab plus pertuzumab combined with toripalimab (a programmed cell death protein 1 [PD-1] inhibitor) immunotherapy. Due to financial constraints, pertuzumab was discontinued after one cycle, and the patient continued trastuzumab plus toripalimab. In December 2023, she received radiotherapy to the right supraclavicular region and right chest wall (50 Gy/25 fractions, with a boost of 16 Gy/8 fractions to the supraclavicular lymph node). In March 2024, she developed myocardial injury, considered to be an immune-related adverse event, leading to discontinuation of toripalimab. Trastuzumab monotherapy was continued.

In June 2024, the patient developed scattered red, pruritic, painful nodules on the right chest wall, which progressively increased in size and number. A skin biopsy of the new lesions was performed in July 2024. Histopathological examination revealed infiltrating poorly differentiated carcinoma in the dermis (Figs. 2C, D). IHC of the metastatic skin lesion showed: ER (focally weakly positive, <1%), PR(−), HER2 IHC 0 (no membrane staining), Ki-67 (~60%+); GCDFP-15 (focal +), GATA3(+), SOX-10 (+) (Fig. 3D–F). Based on the current American Society of Clinical Oncology/College of American Pathologists (ASCO/CAP) guidelines, HER2 status was assessed by IHC. The HER2 IHC 0 status (no membrane staining) confirmed HER2 negativity, excluding HER2-low and HER2-ultralow disease, and thus precluded the use of antibody-drug conjugates such as trastuzumab deruxtecan. These findings confirmed a persistent triple-negative phenotype in the cutaneous metastasis.

Notably, the patient’s laboratory tests, including complete blood count and tumor markers (CEA, CA125, CA15-3), were all within normal limits. Cranial magnetic resonance imaging (MRI), chest computed tomography (CT), and abdominal CT also showed no evidence of distant metastases. This pattern of laboratory and imaging findings underscores the importance of re-biopsying metastatic lesions in breast cancer patients with cutaneous involvement. Such reevaluation is pivotal for guiding subsequent therapeutic adjustments and impacts patient prognosis.

The clinical timeline and management interventions are summarized in Table 1. Following the biopsy-confirmed persistent triple-negative phenotype in the cutaneous metastasis, the treatment regimen was switched to gemcitabine plus cisplatin chemotherapy combined with envafolimab (a PD-L1 inhibitor) immunotherapy. Prior to the initiation of envafolimab, PD-L1 immunohistochemistry (clone SP263, Ventana platform) was performed on the cutaneous metastatic lesion, revealing tumor cell (TC) positivity of 0%, combined positive score for tumor-associated immune cells (IC+) of < 1%, and immune cell proportion (ICP) of approximately 10%. The patient received 6 cycles of gemcitabine plus cisplatin combined with envafolimab between August and November 2024. The treatment course was documented as uneventful, with isolated grade 3 neutropenia (neutrophil count 0.71 × 10^9/L) observed in October 2024. No other severe adverse events were recorded during this period. In January 2025, new cutaneous lesions emerged on the right chest wall, axilla, and back. A repeat biopsy revealed ER 20% positivity with persistent HER2 negativity (HER2 IHC 0), indicating a secondary phenotypic shift. The regimen was subsequently adjusted to vinorelbine plus bevacizumab combined with envafolimab. The disease has been controlled under this regimen, and the patient remains under active follow-up.

**Table 1:** Clinical timeline of diagnosis, treatment, and disease course.

Date/Time	Clinical Event & Management
Jan 2023	A right breast mass was discovered (approximately 1.5 × 1.0 cm).
Apr 2023	Diagnosis: Invasive breast carcinoma. Primary tumor IHC: HER2 (3+), ER/PR (-).
Apr–Sep 2023	Neoadjuvant therapy: Cyclophosphamide + epirubicin (2 cycles); paclitaxel liposome + cyclophosphamide + epirubicin (2 cycles); paclitaxel liposome + trastuzumab + pertuzumab (2 cycles).
Sep 2023	Surgery: Underwent right modified radical mastectomy. Postoperative pathology: ypT2N3aMx (extensive lymph node metastasis, 23/23 positive). Primary tumor IHC: HER2 (3+), ER/PR (-), Ki-67 ~40%.
Sep–Oct 2023	Adjuvant therapy: Paclitaxel liposome + carboplatin combined with trastuzumab + pertuzumab (2 cycles).
Oct 2023	First metastasis: Supraclavicular lymph node metastasis. Pathology confirmed phenotypic conversion to HER2-negative: HER2 IHC 0, ER ~1%. Treatment switched to trastuzumab + pertuzumab + toripalimab.
Nov 2023	Due to financial constraints, pertuzumab discontinued; continued trastuzumab + toripalimab.
Dec 2023	Radiotherapy to right supraclavicular region and chest wall (50 Gy/25F + 16 Gy/8F boost).
Mar 2024	Immune-related myocardial injury; toripalimab discontinued. Continued trastuzumab monotherapy.
Jun 2024	Disease progression: Cutaneous metastasis on the right chest wall identified.
Jul 2024	Skin biopsy confirmed persistent triple-negative phenotype: HER2 IHC 0 (no membrane staining), ER < 1%. PD-L1 (SP263, Ventana): TC 0%, IC+ <1%, ICP ~10%. Treatment switched to gemcitabine + cisplatin + envafolimab.
Aug–Nov 2024	Completed 6 cycles of gemcitabine + cisplatin combined with envafolimab.
Jan 2025	New cutaneous lesions. Biopsy showed secondary phenotypic shift: ER 20%+, HER2 IHC 0. Regimen adjusted to vinorelbine + bevacizumab + envafolimab.
Apr 2025	Last documented follow-up: Patient on active treatment with stable disease status.

ER = estrogen receptor, HER2 = human epidermal growth factor receptor 2, IC+ = positive tumor-associated immune cells, ICP = immune cell proportion, IHC = immunohistochemistry, PD-L1 = programmed death-ligand 1, PR = progesterone receptor, TC = tumor cell positivity.

## 3. Discussion and conclusion

Breast cancer is the most common malignancy in women, and its high degree of heterogeneity dictates variations in treatment strategies and prognosis. Based on the expression of estrogen receptor (ER), progesterone receptor (PR), human epidermal growth factor receptor 2 (HER2), and Ki-67, breast cancer can be classified into molecular subtypes such as Luminal A, Luminal B, HER2-overexpressing, and triple-negative. This classification forms the basis for developing personalized treatment plans.^[6,7]^ However, clinical practice has revealed that in approximately 10–30% of cases, the molecular phenotype may change upon recurrence or metastasis, a phenomenon termed “phenotypic conversion.”^[8–10]^ Phenotypic conversion may involve key biological processes such as epithelial-mesenchymal transition (EMT). However, receptor discordance can also reflect tumor heterogeneity, clonal selection under therapeutic pressure, sampling differences, or technical variability.^[11,12]^ Multiple studies indicate that molecular phenotypic alterations can be observed in nearly half of patients with cutaneous metastases.^[9]^ The most common phenotypic shift is the loss of hormone receptor status, particularly conversion from a hormone receptor-positive primary tumor to a triple-negative metastatic phenotype.^[9]^ Immunohistochemical analyses show discordance in receptor expression between primary and metastatic tumors, which can reach clinically relevant levels.^[9]^ Specifically, conversion from a hormone receptor-positive subtype to the more aggressive triple-negative breast cancer necessitates a fundamental change in treatment strategy and is associated with a worse prognosis.^[8,13–15]^

Regarding molecular subtype distribution, HER2-positive breast cancer is more prone to cutaneous metastasis.^[16]^ Notably, while the HER2-positive subtype is relatively common in cutaneous metastases, some cases exhibit HER2-positive metastases even when the primary tumor was HER2-negative.^[9]^ Furthermore, molecular phenotypic conversion following cutaneous metastasis in breast cancer shows a significant correlation with patient survival rates. Studies demonstrate that patients with metastases exhibiting phenotypic conversion have a markedly poorer prognosis and worse survival outcomes.^[8,17]^ This conversion promotes the formation of distant metastases by enhancing tumor cell migration and invasion capabilities.^[18]^

In this case, the patient initially presented with a HER2-overexpressing primary tumor (HER2 IHC 3+). The first evidence of phenotypic conversion appeared in October 2023, when a supraclavicular lymph node biopsy revealed HER2 IHC 0, consistent with a triple-negative phenotype. This was subsequently confirmed in July 2024 by biopsy of the cutaneous metastatic lesion, which also demonstrated HER2 IHC 0. Notably, the January 2025 biopsy of new cutaneous lesions revealed a further phenotypic shift with ER 20% positivity, suggesting ongoing clonal evolution. Research indicates that abnormal activation of the Wnt/β-catenin signaling pathway may drive the formation of a basal-like breast cancer (BLBC) phenotype,^[19,20]^ and factors such as bone morphogenetic protein-2 (BMP-2) can enhance cancer stem cell properties by promoting EMT.^[21]^ Such phenotypic conversions often lead to treatment resistance,^[22]^ rendering traditional endocrine and targeted therapies ineffective. This finding underscores that re-biopsy of accessible recurrent or metastatic lesions is strongly recommended to reveal the tumor’s clonal evolution and guide precise treatment.^[23]^ Treatment based solely on the primary HER2-positive status may have led to inappropriate continuation of HER2-directed therapy. Once it was confirmed that the metastases had converted to a molecular subtype with triple-negative features, the treatment team opted for a regimen of gemcitabine plus cisplatin combined with envafolimab (a PD-L1 inhibitor). This decision aligns with the current first-line treatment standards for advanced triple-negative breast cancer (TNBC).^[24,25]^

An additional noteworthy finding in this case was the secondary phenotypic shift observed in January 2025, when a repeat biopsy of new cutaneous lesions demonstrated ER 20% positivity with persistent HER2 negativity. This suggests that phenotypic conversion is not a static event but may represent ongoing clonal evolution under therapeutic pressure. There of ER expression, albeit at low levels, further complicates treatment decisions and highlights the necessity of repeated biopsies at each disease progression.

Biomarker-guided therapy: Prior to the initiation of envafolimab, PD-L1 expression was assessed by IHC (clone SP263, Ventana) on the cutaneous metastatic lesion, revealing TC 0%, IC+ <1%, and ICP ~10%. While PD-L1 expression was low, the decision to use envafolimab combined with chemotherapy was based on the established treatment paradigm for advanced triple-negative breast cancer, given the absence of effective targeted alternatives following phenotypic conversion. Although PD-L1 positivity (particularly combined positive score [CPS] ≥ 10) is associated with greater benefit from immunotherapy in TNBC, emerging evidence suggests that some patients with low or negative PD-L1 may still derive clinical benefit. We acknowledge that comprehensive biomarker testing for microsatellite instability/deficient mismatch repair (MSI/dMMR) and tumor mutational burden (TMB) was not performed at our institution.

This case presents the complex clinical course of a high-risk HER2-positive breast cancer that rapidly recurred with conversion to a HER2-negative phenotype following resistance to neoadjuvant chemotherapy. The diagnostic and therapeutic process highlights the necessity of repeated biopsy, emphasizing that for recurrent or metastatic lesions, biomarkers must be reevaluated to reveal the tumor’s clonal evolution and guide precise treatment. The present case further illustrates that phenotypic conversion may first manifest in lymph node metastases before becoming clinically evident in cutaneous lesions, underscoring the importance of systematic biomarker surveillance throughout the disease course.

### 3.1. Patient perspective

The patient was concerned about the skin recurrence. After understanding that the cancer’s phenotype had converted, leading to the treatment change, she consented to and is tolerating the new regimen, with current disease stability.

## Acknowledgments

The authors thank the patient for providing informed consent for publication of this case. We also acknowledge the clinical staff involved in the patient’s care.

## Author contributions

**Data curation:** Shucheng Zhang, Xiaoyue Sun.

**Investigation:** Shucheng Zhang, Xiaoyue Sun.

**Writing – original draft:** Shucheng Zhang.

**Formal analysis:** Zhuqing Li.

**Writing – review & editing:** Zhuqing Li, Shijuan Wei, Xiaoqing Si.

**Conceptualization:** Shijuan Wei, Xiaoqing Si.
